# Perceptions of the Furhat social robot administering a mental health assessment: a pilot mixed-method exploration

**DOI:** 10.3389/frobt.2025.1737028

**Published:** 2026-01-08

**Authors:** Paulina Tsvetkova

**Affiliations:** 1 Institute of Robotics, Bulgarian Academy of Sciences, Sofia, Bulgaria; 2 Faculty of Information Sciences, University of Library Studies and Information Technologies, Sofia, Bulgaria

**Keywords:** competence, discomfort, Furhat robot, human-robot interaction, mental health, perceptions, psychological assessment, warmth

## Abstract

In the era of artificial intelligence and rapidly advancing robotics, the field of Human–Robot Interaction (HRI) has taken center stage across multiple domains, including psychology. From a psychological perspective, it is therefore essential to deepen our understanding of the factors that shape the quality of these interactions and their implications. This emphasis also aligns with the principles of Industry 5.0, which prioritize human well-being and use technologies to promote sustainable progress. The present study employs an exploratory mixed-method approach and aims to examine perceptions of warmth, competence and discomfort with the Furhat social robot in a psychological assessment setting. Specifically, we investigated young adults’ interactions with the Furhat social robot while it administered the Depression, Anxiety and Stress Scale (DASS-21). Following the interaction, the participants completed the short version of the Robot Social Attributes Scale (RoSAS-SF) to assess perceived warmth, competence and discomfort, and provided qualitative feedback regarding their interactional experiences and acceptance of the robot. The findings provide preliminary insights into the respondents’ perceptions of and openness toward robot-administered psychological screening, suggesting that the Furhat social robot may have potential as an assistive tool in mental health assessment contexts. These results highlight the need for further research with larger samples to examine the role of social robots in psychological practice more comprehensively.

## 1 Introduction

Social robotics is focused on creating and deploying robots that can engage with humans in a natural and socially appropriate manner. This is a fast-growing technology used in different fields like education, service and healthcare. Social robots can build trust by offering consistently positive interactions, ensuring smooth and engaging communication across modalities such as speech and gaze (Tsirka et al., 2025). Integrating speech, gestures and facial expressions, social robots and Socially-Assistive Robots (SARs) foster a strong connection with people and encourage self-disclosures (Spitale et al., 2024). Within this context, Human–Robot Interaction (HRI) has become a central area of research, examining how humans perceive, respond to and interact with robots. Robots need to recognize and show emotions, as this enables more natural interactions and enhances both social presence and communication effectiveness (Ottoni and Cerqueira, 2024). In HRI aimed at supporting psychological wellbeing, emotional engagement and user perceptions are crucial factors. An interesting study indicates that robots can support the development of collaborative skills by embedding basic elements of social behavior within game-based interactions (Dimitrova et al., 2012). Another study reveals that, over time, people disclose more to social robots and perceive them as increasingly social and competent, suggesting that social robots can be effective conversational partners in emotional health interventions (Laban et al., 2023).

Robotic psychology is an interdisciplinary field that investigates humans’ emotional, cognitive, social and physical responses to human–robot interactions, taking into account both social and physical environments (Stock-Homburg, 2021). In the psychological domains, a systematic review indicates that social robot–based interventions can effectively enhance three key areas: social functioning, emotional state, and overall wellbeing (such as stress and anxiety reduction), sometimes performing comparably or even better than humans in specific tasks. Most interventions in the psychological domain targeted children with Autism Spectrum Disorder (ASD), although other groups were also addressed (Duradoni et al., 2021).

This interdisciplinary perspective, bridging robotics and psychology, examines not only how robots are designed to behave socially, but also how humans communicate and interact with social robots—including in sensitive settings such as mental health. Mental health disorders–including depression, stress and anxiety - are major causes of health-related issues among young people worldwide. According to an article published on the official site of UNESCO “Across the world, more children and young people are reporting stress, anxiety and emotional distress. One in seven youngsters lives with a diagnosable mental health condition, and many more face difficulties that affect their wellbeing, learning, and futures”^1^. Advances in assistive technologies have the potential to address these challenges by providing innovative tools for support, intervention and assessment. The physical embodiment of social robots and SARs contributes to more meaningful interactions, which is especially important in mental health contexts where trust and empathy are essential (Cano et al., 2021). Recent studies have explored how social robots can assist the psychological wellbeing of children and elderly (Or et al., 2025; López-Durán et al., 2025). Both studies report a reduction in stress, anxiety or depression. Social robots could also enhance digital mental healthcare, if they are able to create meaningful interactions that focus on the user’s needs (Jung et al., 2025). The authors in a review (Jung et al., 2025) observe that effective SARs should foster users’ autonomy, competence and emotional wellbeing as core design goals. These findings suggest that social robots are promising tools for therapeutic purposes (Abbasi et al., 2025).

Studies on psychotherapy and intervention, integrating social robots show very promising outcomes, including improvements in wellbeing and mood of the participants (Chen et al., 2020; Sawik et al., 2023). Some authors suggest that attachment between clients and (robot) therapists is essential for successful therapeutic outcomes. Moreover, social robots can achieve their full potential in mental health applications only when the intensity of attachment corresponds appropriately to the type of relationship formed (Szondy and Fazekas, 2024). Observations and feedback from participants in a systematic review suggested that users found the experience with social robots enjoyable and readily accepted it (Kling et al., 2025).

Despite growing evidence for the effectiveness of social robots in mental health and well-being, research on their use as instruments for psychological assessment remains limited. Preliminary studies suggest that humanoid robots can administer cognitive and behavioral assessments, providing standardized, unbiased and engaging evaluation tools. For example, a pilot study testing a humanoid robot programmed to deliver a cognitive screening tool for Mild Cognitive Impairment demonstrated promising results, showing comparable outcomes to traditional paper-based assessments and highlighting the potential of robots for large-scale cognitive evaluation among older adults (Varrasi et al., 2018). Another study used social robots as diagnostic tools for social anxiety, showing that robot-based behavioral assessments could distinguish individuals with social anxiety disorder from those without it. The authors note that social robots can support clinicians by enhancing interventions for social anxiety, serving as complementary tools rather than replacements in treatment (Rasouli et al., 2022). The main aim of the present study was to examine perceptions of warmth, competence and discomfort toward the social robot Furhat, administering a psychological questionnaire.

## 2 Materials and methods

This study received ethical approval from the Ethics Committee of the Institute of Robotics, Bulgarian Academy of Sciences (Reference: No11/03.10.2025).

### 2.1 The social robot Furhat

Furhat is the most advanced conversational robot capable of human-like interactions, including speech, gestures, facial expressions and emotion expressions^2^. The Furhat robot is currently used in a variety of settings, including psychotherapy training, where it demonstrates feasibility and effectiveness (Johansson et al., 2017). The robot also shows potential as a platform for administering psychological assessments, such as measures of stress, anxiety and depression (Nandanwar and Dutt, 2023). However, that study lacks details on the dialogue flowchart and the programming implementation. To address these gaps, we have developed a Furhat PsychScreen framework for administering different psychological questionnaires using the Furhat robot platform (Lekova et al., 2024). We subsequently tested the framework and the preliminary results are described in the present study, in which the robot administered a mental health assessment in the participants’ native language. The framework allows customization of items, scales, scoring and response formats. It combines voice-based interaction with a touchscreen Graphical User Interface (GUI), enabling participants to respond through dialogue with the robot or via a traditional interface. The dialogue is managed via hierarchical state diagrams implemented in a human-readable Kotlin-based Domain-Specific Language (DSL). The main component of the framework is the Flow, managing user interactions, speech and gestures. Other key components include NLU-based Intents and Entities, NLU Assets for speech parsing and a User Manager tracking participant presence.

### 2.2 Mixed-method approach

The study employed a mixed-method approach, incorporating both quantitative and qualitative data. Due to the small sample size, the analysis was planned as descriptive and exploratory. Summary statistics were used to evaluate participants’ perceptions of the robot. On the other hand, the qualitative findings followed a descriptive approach and complemented the quantitative measures, thus providing a deeper understanding of the attitudes reported by the young people. Furhat administered a validated and standardized psychological questionnaire- DASS-21, a shortened version of the DASS-42, which assesses three subscales–depression, anxiety and stress. Initial evidence supports its adequate convergent and discriminant validity (Lovibond and Lovibond, 1995). The questionnaire comprises 21 items, with seven items assessing each of the three subscales over the past week. Each statement is rated on a 4-point scale from 0 (“did not apply at all”) to 3 (“applied very much”). Subscale totals are derived by adding item scores, providing an overall assessment of symptom severity. Although administered to the participants, DASS-21 scores were not included in the quantitative analysis, as the focus of the present study was on participants’ perceptions of the robot rather than their psychological condition.

The quantitative analysis included the participants’ perceptions of the social robot via the short version of the RoSAS questionnaire - Robot Social Attributes Scale (Carpinella et al., 2017) which was administered post-interaction. The short form of the RoSAS (RoSAS-SF) is a six-item scale designed to assess attitudes toward robots in three dimensions: warmth (e.g., compassionate, sociable), competence (e.g., competent, reliable) and discomfort (e.g., scary, awkward) (Neuenswander et al., 2025). These dimensions provide insights into participants’ attitudes toward the robot, which are key indicators of trust and willingness to engage with it.

In terms of the qualitative aspect of the analysis, it followed a qualitative description approach which describes phenomena rather than explains them^3^. This approach helped us contextualize participants’ perceptions of the robot and complemented the quantitative findings. In addition to the quantitative measures, a qualitative analysis was conducted to explore respondents’ feedback and to obtain a better idea of their interaction with the robot. The young adults responded to the following open-ended questions:

- “Did you feel engaged in your interaction with the robot?”;
- Will you trust the robot to administer another questionnaire to you ?” (Please explain why or why not.);
- “What aspects of the robot’s behavior and appearance did you dislike?”;
- “Do you have any suggestions for improving the robot or its interaction style?”

### 2.3 Participants

The experiment included ten young adults from the general public (five men and five women, mean age 22). Half of them are undergraduate and the other half are postgraduate. The participants were selected randomly from a group of individuals previously taught by the researcher. They were invited to participate through individual phone calls and in-person meetings. All individuals who were approached agreed to take part in the study. Nine participants stated having intermediate experience with technology, and one participant noted basic experience. None of them has interacted with social robots before. All respondents self-reported a normal mental health status. Although cultural background was not within the scope of the present study, the participants were recruited from the same geographical area and were enrolled in universities within the same region. This suggests a relatively homogeneous cultural context and reduces the likelihood of substantial cultural differences that could influence the results.

### 2.4 Experimental setup

**FIGURE 1:**
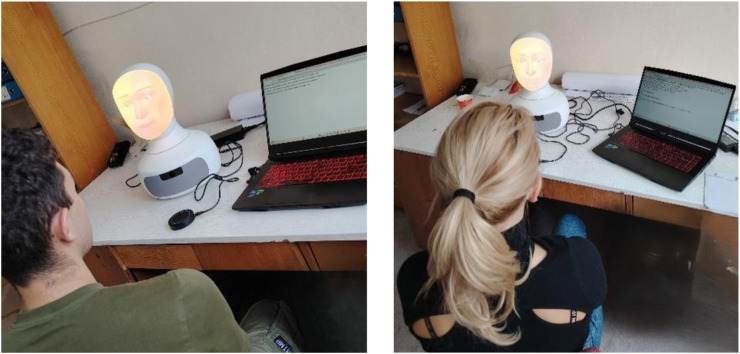
Experimental setup with participants.

The study was conducted in a quiet room at the Institute of Robotics, Bulgarian Academy of Sciences, to provide a controlled and calm environment for participant–robot interaction. The participants were seated approximately 1 m from the Furhat social robot, facing it directly. The robot was equipped with a microphone and a screen to facilitate verbal communication and to display the questionnaire statements as well as the response scale. Furhat could also repeat the statements or the response scale when needed. Using the Furhat PsychScreen Framework (Lekova et al., 2024), the robot administered the DASS-21 questionnaire in the participants’ native language through Microsoft Azure services. Each participant responded verbally to the 21 statements, with the robot following a predefined dialogue sequence and logging responses. Subscale scores were automatically calculated by summing item scores, providing overall measures of depression, anxiety and stress. A psychologist, trained to operate the robot, was present during the sessions to observe and manage the process. Although this may have caused social desirability bias to some extent, the risk was reduced by ensuring that the psychologist did not interact with the participants and maintained a non-intrusive presence. The psychologist also administered a short version of the RoSAS questionnaire post-interaction and interviewed the respondents to assess their attitudes and perceptions toward the robot. Every session lasted approximately 15 min in individual formats. The quantitative data from the RoSAS were analyzed using Python to compute descriptive statistics, while qualitative responses to the four open-ended questions were examined through manual thematic analysis to identify recurring themes related to trust, engagement and interactional challenges. Photos of the sessions can be seen in Figure 1.

## 3 Results

### 3.1 Quantitative results: RoSAS data analysis

**FIGURE 2:**
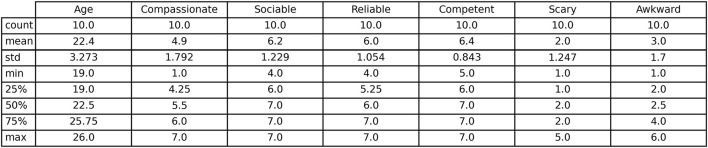
Descriptive statistics of Rosas.

Given the exploratory nature of the study and the small sample size, the quantitative results are reported descriptively and illustrate preliminary patterns in HRI. First, Furhat administered the DASS-21 questionnaire to the young adults. They were asked to respond verbally on a 4-point scale. For the purpose of the current study, their responses to the mental health assessment were not analyzed. After the interaction, the respondents evaluated the robot using the RoSAS scale and were interviewed by the psychologist for qualitative data. The scores for the six RoSAS adjectives were processed and analyzed to examine how participants rated each of them. Descriptive statistics were used to summarize participants’ assessments both at the item level (i.e., the six individual adjectives) and at the dimension level, corresponding to the three RoSAS dimensions: warmth, competence and discomfort. Given that 4 represents the midpoint of the 7-point Likert scale used in the RoSAS measure, values above 4 for the positive valence adjectives (compassionate, reliable, sociable and competent) indicate more positive perceptions, whereas higher scores for the negative valence adjectives (scary and awkward) reflect more negative perceptions. The results showed that the mean values of the four positive adjectives (compassionate, sociable, reliable and competent) were above 4, with “competent” and “sociable” receiving the highest ratings (6.4 and 6.2, respectively). In contrast, the adjectives “scary” and “awkward” were rated under 4 (2.0 and 3.0), revealing lower perceived discomfort within this sample. Overall, these exploratory results suggest that overall, the robot was perceived as capable and that the respondents found it easy to engage with. The standard deviations showed that participants’ views were most consistent regarding the adjectives “competent” and “reliable” (SDs between 0.84 and 1.05), suggesting relatively consistent opinions that the robot performed the task efficiently. A table of the descriptive statistics can be seen in Figure 2.

**FIGURE 3:**
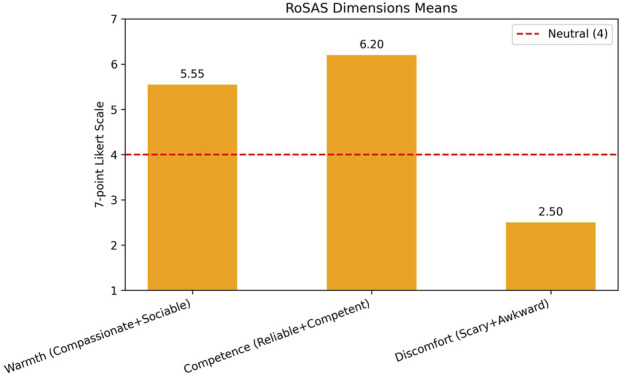
Mean scores for warmth, competence and discomfort dimensions of the RoSAS.

The mean scores across the three RoSAS dimensions (Warmth, Competence and Discomfort) indicate that the participants rated the robot highly on Competence (M = 6.20) and Warmth (M = 5.55), both above the neutral midpoint of 4. The high scores for Competence suggest that the respondents trust the robot’s performance. On the other hand, Discomfort received a low mean score (M = 2.50), suggesting that the young adults tended to evaluate Furhat as more competent and friendly than discomforting. Within this sample, the individuals perceived the robot as trustworthy and they indicated feeling at ease during the interaction with it. In this exploratory study, focusing on participants’ individual responses allowed us to capture nuances in trust and engagement that may not be evident in group-level analyses and that are central to psychotherapeutic settings. The mean scores for each of the three dimensions of RoSAS are visualized in Figure 3.

### 3.2 Qualitative results: descriptive qualitative analysis

In terms of the qualitative data, we applied a qualitative descriptive design. This type of approach requires a level of interpretation, which should remain closely aligned with participants’ descriptions, without moving beyond the data (Sandelowski, 2010; Bradshaw et al., 2017). A well-conducted qualitative descriptive study provides a detailed summary of an event, reflecting what it means to those who experienced it (Sandelowski, 2000). In the present study the descriptive qualitative analysis aimed to summarize the respondents’ reflections and to provide contextual insights into their perceptions of the robot.

Following this qualitative descriptive approach, our analysis focused on each participant’s response to four open-ended questions addressing trust, engagement and interactional challenges. Regarding the first two questions “Did you feel engaged in your interaction with the robot?” and “Will you trust the robot to administer another questionnaire to you”, all participants reported feeling engaged and admitted they enjoyed interacting with the robot. Several respondents characterized the interaction as “pleasant” and “interesting” and all of them shared that they would trust the robot to administer other psychological questionnaires. The respondents emphasized that the robot’s calm voice and nonjudgmental manner helped them feel at ease and contributed to their willingness to respond openly to the DASS-21 items. Some individual differences were also reflected in the responses. One participant (a female) noted that she still felt some prejudice about speaking to a machine rather than a person, which prevented her from fully confiding in it. Another respondent mentioned that sharing a difficult situation with the robot could be “easier than talking to a psychologist” because she would not feel evaluated or ashamed. Overall, the participants’ descriptions of the robot as approachable appeared to encourage openness during the screening. These qualitative observations align with the high ratings of the adjectives “reliable”, “sociable” and “competent” as well as the low ratings of “scary” and “awkward” in the quantitative analysis.

The respondents described several challenges encountered during the interaction with the robot, particularly related to communication and social expressivity. In response to the next question, “What aspects of the robot’s behavior and appearance did you dislike?”, a few participants reported occasional difficulties with speech recognition, noting that the robot sometimes failed to accurately register their verbal responses, requiring them to repeat or rephrase their answers. For example, several participants mentioned that the robot very often misinterpreted the number “0” (pronounced “nula” in Bulgarian), leading to minor frustration during the screening. In addition, some participants indicated that the robot’s limited facial expressivity and nonverbal communication reduced the perceived naturalness of the interaction. Those young people pointed out that they would have liked the robot more if it had shown more nonverbal behavior. They emphasized that richer facial expressions, gestures and a warmer tone would make the robot appear more engaging and emotionally responsive. These findings complement the relatively neutral quantitative ratings for the adjective “compassionate”, suggesting mixed perceptions of the robot’s warmth. It is worth noting, however, that administering a psychological questionnaire requires a neutral tone and limited expressivity, in contrast to the dynamic turn-taking typical of real-time social interactions with a psychologist. Despite the minor limitations, the participants’ overall evaluation remained positive and many of them expressed willingness to engage with the robot in similar interactions in the future.

In response to the question „Do you have any suggestions for improving the robot or its interaction style?“ the participants suggested incorporating more lifelike eye movements and even a more human-like appearance. These recommendations could be directed to the robot’s manufacturers, as implementing them would require modifications to the hardware design. The respondents also expressed curiosity about more conversational or interactive exchanges, indicating that future versions of the system could support open-ended dialogue.

Overall, the drawbacks observed in the participant-robot interaction were related to occasional speech recognition errors and limited facial expressions. Other psychological factors that contribute to people’s willingness to interact with social robots need to be explored in depth, too. Individuals’ eagerness to engage with such technology can also be influenced by various intrapersonal characteristics, such as openness to new technologies, prior experience with robotic systems, trust in technical devices and hedonistic motivation, as well as external factors, including age and the environmental conditions in which interactions occur (Saenko et al., 2025).

Taken together, the qualitative findings suggest that the respondents were receptive to robot-mediated psychological screening and tended to describe the robot as trustworthy and reliable. These patterns are consistent with the quantitative RoSAS results, which showed high ratings for Warmth and Competence and low ratings of Discomfort within this sample. These results provide a foundation for future studies exploring robot-mediated psychological screening with larger and more diverse participant groups and employing more systematic qualitative methodologies.

## 4 Discussion and limitations

The findings of this exploratory study suggest that the robot may be perceived as approachable, fostering generally positive perceptions and initial trust among participants. These findings align with previous research suggesting that social robots can provide a sense of psychological comfort and safety in sensitive contexts, such as self-disclosure or disclosing distress (Laban et al., 2025). However, given the limited sample size, the quantitative and qualitative analyses were used to identify preliminary trends in participants’ perceptions of the Furhat social robot, rather than to draw generalizable conclusions and to establish validity. Larger-scale studies will be necessary to provide more comprehensive analyses and to validate robot-administered assessments.

A key limitation of the present study is the small sample size (N = 10), which limits the extent to which conclusions about perceptions of warmth, competence and discomfort toward the robot can be generalized. Another limitation is the lack of a control condition for direct comparison with traditional assessment methods. The focus of the current work was on an exploratory analysis of the individuals’ perceptions of interacting with a social robot in a mental health screening context. The findings, therefore, offer initial insights into young people’s trust and openness to sharing personal information with such technology.

Although based on a small sample, the observations suggest that the young adults may be open to robot-mediated psychological screening, highlighting the potential of using such technology in psychological practice. These results provide preliminary psychological insights to the growing research suggesting that social robots may play a supportive role in psychological settings. By creating a nonjudgmental space for communication, robots like Furhat may help young people express themselves more openly, offering new opportunities for engagement in mental health and wellbeing interventions.

Planned subsequent studies will extend the sample size and investigate the perceptions of warmth, competence and discomfort in greater depth, providing more robust and generalizable conclusions. Furthermore, future research should focus on improving speech recognition and enhancing the robot’s emotional expressivity. In addition, direct comparisons between robot-administered and paper-based assessments will also be essential for establishing psychometric reliability and contextual factors influencing trust and engagement. Such research will contribute to the broader applicability and long-term integration of social robots in the psychological domain.

## Data Availability

The original contributions presented in the study are included in the article/supplementary material, further inquiries can be directed to the corresponding author.

## Footnotes

1
https://www.unesco.org/en/health-education/mental-health

2
https://www.furhatrobotics.com/

3
https://oercollective.caul.edu.au/qualitative-research/chapter/5/
